# Efficacy and safety of additional surgery after non-curative endoscopic submucosal dissection for early colorectal cancer

**DOI:** 10.1186/s12876-017-0701-y

**Published:** 2017-11-28

**Authors:** Tao Chen, Yi-Qun Zhang, Wei-Feng Chen, Ying-Yong Hou, Li-Qing Yao, Yun-Shi Zhong, Mei-Dong Xu, Ping-Hong Zhou

**Affiliations:** 1 Endoscopy center, Zhongshan Hospital, and Endoscopy Research Institute, Fudan University, Shanghai, 200032 China; 2 Department of Pathology, Zhongshan Hospital of Fudan University, Shanghai, 200032 China

**Keywords:** Early cancer, Colorectum, Endoscopic submucosal dissection, Additional surgery

## Abstract

**Background:**

Additional surgery is recommended when early colorectal cancer (ECRC) is resected by non-curative endoscopic submucosal dissection (ESD) and there is significant risk of lymph node metastasis (LNM). The aim of this study was to investigate the efficacy and safety of additional surgery after non-curative ESD for ECRC and evaluate long-term outcomes.

**Methods:**

Patients with ECRC who underwent ESD and additional surgery between July 2007 and November 2013 were identified. Histology and patient data were collected during an average period of more than 5 years to determine tumor stage and type, resection status, complications, tumor recurrence, and distant metastasis.

**Results:**

Fifty-one patients who underwent additional surgery were eligible for analysis. Overall, regional LNM was detected in 5 patients (9.8%) and presence of lymphovascular infiltration was a significant risk factor. Surgery-related complications occurred in 3 patients (5.9%). During a median follow-up period of 59 months, no metastasis or local recurrence was observed. Three patients died of other diseases and no CRC-related deaths took place.

**Conclusions:**

Additional surgery after non-curative ESD for ECRC is effective and safe and should be encouraged to foster curative treatment and better long-term outcomes.

## Background

Endoscopic submucosal dissection (ESD) is the standard treatment for early colorectal cancers (ECRCs) that have negligible risk of lymph node metastasis (LNM) [1]. Studies have reported that ESD provides high *en bloc* resection rates, which contributes to accurate histopathological evaluation [2, 3]. In addition, ESD provides high R0 resection rates to minimize risk of recurrence. However, curative resection can be evaluated successful only after the patient meets the pathological low-risk criteria, including submucosal invasion of less than 1000 μm, exclusion of poor differentiation, exclusion of lymphovascular infiltration, and no tumor budding [4]. When one of these criteria is not met, additional surgery with lymph node dissection is recommended to prevent lymph node or distant metastasis. To date, few data are available regarding the safety of additional surgery after non-curative ESD and the long-term outcomes. Thus, we investigated the efficacy and safety of additional surgery after non-curative ESD for ECRC and examined its long-term outcomes with an average period of more than 5 years.

## Methods

### Study population and data collection

Between July 2007 and November 2013, a total of 51 ECRCs in 51 patients who had additional surgery after non-curative ESD were identified and analyzed in this study. ESD is indicated for lesions requiring endoscopic *en bloc* resection for which it is difficult to use the snare technique, including laterally spreading tumor (LST), superficial invasive submucosal cancer, large depressed tumors, and large elevated lesions that are likely malignant. Curative resection is defined as submucosal invasion of less than 1000 μm with exclusion of poor differentiation, exclusion of lymphovascular infiltration, and no tumor budding [4]. In addition, piecemeal resection is the critical risk factor for local recurrence. When one of these criteria is not met, additional surgery is recommended. The study was performed in accordance with the Declaration of Helsinki. All patients were informed of the benefits of ESD and the risks of additional surgery, and each patient provided written informed consent for the procedure. This study protocol was approved by the Institutional Review Board of Zhongshan Hospital (No.2009135).

### Treatment for ECRC

ESDs were performed with patients under intravenous anesthesia. Additional surgeries were performed with patients under general anesthesia. Indices of the patients’ cardiorespiratory functions (heart rate, blood pressure, and oxygen saturation) were continually monitored. Endoscopic equipment and accessories were introduced in previous reports [5, 6]. Some colorectal tumors were resected using a combined endoscopic mucosal resection (EMR) and ESD technique (hybrid ESD), with a special ESD knife, and resection was completed by snaring [7]. Some degree of submucosal dissection was involved in hybrid ESD after marginal pre-cutting [1]. When ESD was revealed to be non-curative, patients were referred to surgeons for consultation. For patients who agreed, additional radical surgery was performed in the same manner as initial standard surgery.

### Histopathological evaluation

Resected specimens were stretched, pinned, fixed in formalin solution, and assessed microscopically. The WHO classification for tumors of the digestive system was used for histopathological evaluation [8]. An *en bloc* resection was defined as an excision of the tumor in one piece without fragmentation. Histologic complete resection was defined as *en bloc* resection with negative vertical and lateral margins. Curative resection was defined as a histologic complete resection with no risk of LNM as indicated by histopathological examination of the resected specimen according to the criteria issued by the Japanese Society for Cancer of the Colon and Rectum (JSCCR) [9].

### ESD-related complications

Minor bleeding occurred in most cases. It was treated with immediate coagulation. Delayed bleeding was defined as hematochezia or melena requiring an endoscopic hemostatic procedure from 0 to 14 days after ESD completion. Perforation was classified as a colorectal wall penetration detected during or after ESD procedure.

### Follow-up

All patients were followed up either at our institution or in partnership with their referring institutions. Follow-up colonoscopy was performed at 6 and 12 months after the operation and then annually in 5 years. Abdominal Computed Tomography (CT) was carried out annually. To analyze long-term outcomes, a questionnaire was sent to patients who had no follow-up medical records at our institution or at our partner centers.

### Statistical analysis

Clinicopathological and endoscopic data, including age, sex, tumor characteristics, histology, resection rates, complications, local recurrence, metastasis, and survival rates were collected and analyzed. Statistical analysis was performed using a chi-squared test or Fisher’s exact test as appropriate. Commercial software (IBM SPSS Statistics 18; SPSS, Chicago, IL, U.S.) was used for statistical analysis (*P* < 0.05 was considered statistically significant).

## Results

### Baseline characteristics of patients and tumors

In this retrospective study, we identified and analyzed 51 ECRC treated by ESD in 51 patients, including 31 men and 20 women. The mean age of the patients was 63.0 ± 9.5 years; 56.9% (29/51) of the patients were older than 60 years. The mean tumor size was 2.6 ± 0.9 cm (median, 2.0 cm; range, 1.5–5.0 cm). Among these tumors, 42 tumors (82.4%) were smaller than 4.0 cm, and the other 9 tumors (17.6%) were larger than 4.0 cm. Tumor location included 7 in the right colon (13.7%), 14 in the left colon (27.4%), and 30 in the rectum (58.9%). Macroscopic types included 31 polypoid tumors (60.8%) and 20 LSTs (39.2%). Patient and tumors details are shown in Table 1.

**Table 1:** Baseline characteristics of the patient and tumors

Variables	Total
Patients	51
Age	
Average ± s.d.(median, range),years	63.0 ± 9.5(63,38–80)
≤ 60 years	22(43.1%)
> 60 years	29(56.9%)
Sex	
Male	31(60.8%)
Female	20(39.2%)
Tumors	51
Tumor size	
Average ± s.d.(median, range),cm	2.6 ± 0.9(2.0,1.5–5.0)
< 40 mm	42(82.4%)
≥ 40 mm	9(17.6%)
Tumor location	
Right side of colon	7(13.7%)
Left side of colon	14(27.4%)
Rectum	30(58.9%)
Growth type	
Laterally spreading tumor	20(39.2%)
Polypoid	31(60.8%)

### Outcomes of ESD for ECRCs

Resection methods included 41 cases of ESD (80.4%) and 10 hybrid ESD (19.6%). *En bloc* resection was achieved in 46 of the 51 treated tumors (90.2%), histological complete resection was achieved in 34 cases (66.7%). Histologically, of the 51 tumors, there were 15 (29.4%) T1 cancers with superficial submucosal invasion (<1000 μm) and 36 (70.6%) T1 cancers with deep submucosal invasion (≥1000 μm). Specifically, there were 5 cancers (9.8%) with invasive lymphovascular infiltration. In the present study, 2 patients had perforations that were closed by clips during the ESD procedure and no patient had delayed bleeding. There were no treatment-related deaths. Outcomes related to ESD for ECRCs are shown in Table 2.

**Table 2:** Outcomes of ESD for 51 early colorectal cancers

Variables	Total
Number of tumors	51
Resection Method	
ESD	41(80.4%)
Hybrid ESD	10(19.6%)
Resection status	
En bloc	46(90.2%)
Piecemeal	5(9.8%)
Histological complete resection	
Complete	34(66.7%)
Incomplete	17(33.3%)
Histology	
T1 cancer (<1000 μm)	15(29.4%)
T1 cancer (≥1000 μm)	36(70.6%)
Differentiation	
Well and moderate	49(96.1%)
Poor	2(3.9%)
Lymphovascular infiltration	
Absence	46(90.2%)
Presence	5(9.8%)
Complications	
Perforation	1(2.0%)
Delayed bleeding	0(0.0%)

### Outcomes of additional surgery after colorectal ESD

The average hospital stay after additional surgery was 7.4 ± 3.1 days (median, 6; range, 4–21). There was 1 patient with postoperative heart failure, 1 patient with postoperative anemia, and 1 patient with postoperative urinary tract infection. Pathological examination after additional surgery found 5 patients with regional LNM (9.8%). In this study, lost to follow-up occurred in 2 patients (3.9%) and the follow-up rate was 96.1% (49/51). The overall median follow-up period was 59 months (average 63.9, range 30–115 months). During the follow-up period, no local recurrence was detected. Furthermore, no patients developed metastasis to either the lymph nodes or distant organs. Three patients died of other diseases and no CRC-related death was found. These data appear in Table 3.

**Table 3:** Outcomes of additional surgery after colorectal ESD

Variables	Total
Number of patients	51
Postoperative hospital stay	
Average ± s.d.(median, range), day	7.4 ± 3.1(6,4–21)
Complications	3(5.9%)
Postoperative heart failure	1(2.0%)
Anemia	1(2.0%)
Urinary tract infection	1(2.0%)
Pathological examination	
Lymph node metastasis	5(9.8%)
Follow up	
Lost to follow up	2(3.9%)
Average(median, range), month	63.9(59, 30–115)
Recurrence	0(0.0%)
Metastasis	0(0.0%)
Overall survival	48/51(94.1%)

### Association of the clinicopathological characteristics of ECRCs with regional LNM

In this study, reasons for additional surgery after ESD for ECRCs included incomplete resection (17 cases, 33.3%), positive margin (13 cases, 25.5%), T1 cancers (≥1000 μm, 34 cases, 66.7%), lymphovascular infiltration (5 cases, 9.8%), and poor differentiation (2 cases, 3.9%, Table 4). Of note, 2 patients who had lymphovascular infiltration had deep T1 cancers and 1 patient had positive margin. On univariate analysis, presence of lymphovascular infiltration had significant association with LNM in patients with ECRCs treated by non-curative ESD (*P* = 0.005). No other clinicopathological characteristics were found to be associated with regional LNM (Table 5).

**Table 4:** Reasons of additional surgery after ESD

Variables	Total
Number of tumors	51
Piecemeal resection	5(9.8%)
Positive margin	13(25.5%)
Vertical	11(21.6%)
Circumferential	2(3.9%)
T1 cancer (≥1000 μm)	34(66.7%)
Poor differentiation	2(3.9%)
Lymphovascular infiltration	5(9.8%)

**Table 5:** The association of the clinicopathological characteristics of 51 early colorectal cancers with lymph node metastasis

Factors	Lymph node metastasis	*P* value
Age		
≤ 60 years	3/22 (13.6%)	0.638
> 60 years	2/29(6.9%)	
Sex		
Male	3/31(9.7%)	1.000
Female	2/20(10.0%)	
Tumor size		
< 40 mm	5/42(11.9%)	0.571
≥ 40 mm	0/9(0.0%)	
Tumor location		
Rectum	4/30(13.3%)	0.391
Colon	1/21(4.8%)	
Growth type		
Polypoid	4/31(12.9%)	1.000
LST	1/20(5.0%)	
Resection Method		
ESD	5/41(12.2%)	0.569
Hybrid ESD	0/10(0.0%)	
Resection status		
En bloc	5/46(10.9%)	1.000
Piecemeal	0/5(0.0%)	
Histological complete resection		
Complete	4/34(11.8%)	0.654
Incomplete	1/17(5.9%)	
Histology		
T1 cancer (<1000 μm)	1/15(6.7%)	1.000
T1 cancer (≥1000 μm)	4/36(11.1%)	
Differentiation		
Well and moderate	1/49(2.0%)	0.078
Poor	1/2(50%)	
Lymphovascular infiltration		
Absence	2/46(4.3%)	0.005
Presence	3/5(60.0%)	

## Discussion

Additional surgery is recommended when ECRCs are resected by non-curative ESD and LNM risk exists. Sometimes, additional surgery after endoscopic resection (ER) was reported not to be performed because of high surgical risk including comorbidities and old age [10, 11]. The present study showed that additional surgery after non-curative ESD for ECRCs was safe. Postoperative complications occurred only in 3 patients (5.9%). All of the patients with complications were successfully taken care of using conservative treatments and there were no perioperative deaths. In this study, regional LNM was observed in 5 patients (9.8%). Compared the risk of LNM to the rate of complications, patients benefitted from additional surgery after non-curative ESD. The average postoperative hospital stay was 7.4 days that was comparable to direct surgery. The long-term outcomes were also excellent and no patients developed metastasis to either the lymph nodes or distant organs. The overall survival rate was up to 94.1% and no CRC-related deaths took place during follow-up, which lasted more than 5 years on average.

Currently, indications for ER of early gastrointestinal epithelial cancers have been expanded when no risk of LNM exists. Patients with T1-stage ECRC have LNM in 6–16% of cases, which means that most patients with ECRC do not have metastasis [12]. However, when LNM exists, the cancer cannot be curatively resected by ER alone and requires lymphadenectomy. In addition, piecemeal resection is the critical risk factor for local recurrence, regardless of the ER method used [1]. National Comprehensive Cancer Network (NCCN) guidelines recommend follow-up for T1-stage CRC following ER in a single specimen (*en bloc*), negative vertical and lateral resection margins, histological grade 1 or 2, and without lymphatic and vascular invasion [13]. Submucosal invasion ≥1000 μm, presence of lymphovascular infiltration, poor differentiation, tumor budding, and incomplete resection are independently associated with increased risk of LNM and residual cancer. Therefore, a radical resection is warranted. In this study, the most common reason for additional surgery after ESD was T1-stage cancer with submucosal invasion ≥1000 μm. Kikuchi et al. divided the submucosa (SM) into SM1, SM2, and SM3 and indicated that the cancer with the depth of submucosal invasion had a risk of LNM of 0%, 10%, and 25%, respectively [14]. Thus, additional surgery is essential for these patients with SM2 and SM3 cancers (≥1000 μm).

In the present study, lymphovascular infiltration (5 cases, 9.8%) was also an important reason for additional surgery after non-curative ESD. Kim and his colleagues reported that lymphovascular infiltration was independently associated with LNM [15]. The association between lymphovascular infiltration and regional LNM is consistent with previous studies of factors related to LNM. However, poor differentiation and submucosal invasion depth were not statistically significantly related to LNM, which was probably due to a relatively small number of cases in this study.

The limitations of this study are its retrospective design and relatively small number of study cases. We also did not compare these values to those of patients who underwent surgery initially after diagnosis. Thus, a prospective, randomized controlled study must be performed in the future to validate the results observed.

## Conclusions

Additional surgery after non-curative ESD for ECRC is effective and safe. Patients who underwent additional surgery had favorable long-term outcomes. Additional surgery should be encouraged after non-curative endoscopic resection to foster curative treatment and better long-term outcomes.

## Abbreviations

CT: Computed Tomography
ECRC: Early colorectal cancer
EMR: Endoscopic mucosal resection
ER: Endoscopic resection
ESD: Endoscopic submucosal dissection
JSCCR: Japanese Society for Cancer of the Colon and Rectum
LNM: Lymph node metastasis
LST: Laterally spreading tumor
WHO: Word Health Organization
